# Exosomes and the Future of Immunotherapy in Pancreatic Cancer

**DOI:** 10.3390/ijms20030567

**Published:** 2019-01-29

**Authors:** Ines A. Batista, Sonia A. Melo

**Affiliations:** 1 Instituto de Investigação e Inovação em Saúde, Universidade do Porto, Portugal (i3S), 4200-135 Porto, Portugal; ibatista@ipatimup.pt; 2 Institute of Molecular Pathology & Immunology of the University of Porto (IPATIMUP), 4200-135 Porto, Portugal; 3 Medical Faculty of the University of Porto (FMUP), 4200-319 Porto, Portugal

**Keywords:** pancreatic cancer, exosomes, immunotherapy

## Abstract

Pancreatic ductal adenocarcinoma (PDAC) is a devastating disease, associated with a late diagnosis and a five-year survival rate of 8%. Currently available treatments fall short in improving the survival and quality of life of PDAC patients. The only possible curative option is still the surgical resection of the tumor. Exosomes are extracellular vesicles secreted by cells that transport proteins, lipids, and nucleic acids to other cells, triggering phenotypic changes in the recipient cells. Tumor cells often secrete increased amounts of exosomes. Tumor exosomes are now accepted as important players in the remodeling of PDAC tumor stroma, particularly in the establishment of an immunosuppressive microenvironment. This has sparked the interest in their usefulness as mediators of immunomodulatory effects for the treatment of PDAC. In fact, exosomes are now under study to understand their potential as nanocarriers to stimulate an immune response against cancer. This review highlights the latest findings regarding the function of exosomes in tumor-driven immunomodulation, and the challenges and advantages associated with the use of these vesicles to potentiate immunotherapy in PDAC.

## 1. Introduction

Pancreatic ductal adenocarcinoma (PDAC) is the seventh deadliest cancer worldwide [1,2]. Even though pancreatic cancer is only the twelfth most common cancer [1,3], nearly 460,000 new cases and 430,000 pancreatic cancer-related deaths are estimated per year worldwide [1,2], and numbers that are expected to almost double by 2040 [1]. In fact, PDAC patients face the devastating reality of a five-year survival rate of 8% [4,5,6]. This alarming scenario is attributed to an often late-stage diagnosis, high metastatic potential, and poor response to the currently available treatments [3,7,8]. Surgical resection of the tumor is still the only hope for these patients to achieve a long-term survival. However, only 20% of PDAC patients present resectable tumors as a consequence of a diagnosis at advanced stages of the disease [8]. Furthermore, a high percentage of patients that undergo surgical resection suffer recurrence [9], which results in death within two years [10,11]. The chemotherapeutics currently considered as standard of care in PDAC include gemcitabine, that can be administrated alone or in combination with the therapeutic protocol FOLFIRINOX (i.e., a combination of the drugs eucovorin, 5-fluorouracil, irinotecan, and oxaliplatin), and ABRAXANE (albumin-bound paclitaxel, also known as nab-Paclitaxel). Even though these lead to some degree of improvement in the survival of patients, it is debatable whether such benefits include gain in the patients’ quality of life [8].

PDAC is characterized by the presence of an immunosuppressive environment [12]. Several studies have made contributions to a better understanding of the immune landscape of this tumor, but much is still open for clarification. Clark et al. [13] showed that immunosuppressive cells (i.e., regulatory T cells (TReg cells), tumor-associated macrophages, and myeloid-derived suppressor cells (MDSCs)) are widely present in the early stages of the disease. The development of specific inhibitors of this immunosuppressive response has the potential to bring great improvements for PDAC patients. With the objective of stimulating an immune response against PDAC cells, different types of vaccines and other immunotherapy drugs are under study [12,14]. However, immunotherapy has thus far revealed to be unsuccessful in PDAC patients, when using monoclonal antibodies against CTLA4 and PD-L1 [15,16,17,18], which achieved remarkable results on other solid tumors [19]. Recent efforts have focused on the combination of these immune checkpoint therapies with various treatment approaches, including commonly used chemotherapy drugs and anti-cancer vaccines [20]. 

Exosomes, which are small endosomal-derived vesicles of 30–150 nm that are secreted by most cells to the extracellular space, can enter the blood stream and travel to distant organs and tissues [21,22]. Exosomes carry proteins, lipids, RNA, and DNA and function as mediators of cell–cell communication [21,22,23,24]. The past years have seen little to no advances in the development of new and more effective treatments for PDAC patients. Recently, exosomes were uncovered as potential tools for the delivery of chemotherapy, antigens, and immunotherapy drugs to cancer cells [25,26,27]. With this review, we aim to highlight the potential of using exosomes to stimulate the immune system of PDAC patients. We describe the known functions of pancreatic cancer exosomes in immunosuppression, and point out how PDAC patients can benefit from this approach to trigger the elimination of pancreatic cancer cells by immune cells. 

## 2. Biogenesis of Exosomes

Exosomes were first described in 1983 [28] when Pan and Johnstone reported that reticulocytes release transferrin receptors (TFR) through small vesicles into the extracellular environment. It was then suggested that this process was necessary for reticulocytes to dispose of this transmembrane receptor during their maturation. This opened a whole new world of possibilities regarding the way cells communicate with each other to induce changes in distant cells. In fact, the rapid evolution of the field of exosomes biology and the discovery of several pathways and phenotype changes that are regulated by intercellular communication via exosomes have encouraged researchers to explore exosomes as tools for the treatment of human diseases, including cancer. Exosomes are defined as small extracellular vesicles of endosomal origin, whose size falls between 30 and 150 nm. These vesicles carry diverse cellular contents that mirror the cell of origin, including proteins, lipids, miRNA, mRNA, and DNA [29,30]. Therefore, it has become evident that cancer-derived exosomes are useful for the diagnosis and prognosis of cancer, allowing to indirectly access the mutational profile [31,32] as well as mRNA, microRNA and protein expression patterns of cancer cells [33,34]. Exosomes are released by most cell types into the extracellular environment, being able to either target neighbor cells or travel to distant organs and tissues. Exosomes then enter a recipient cell and release their cargo to regulate protein expression and diverse signaling pathways [21,22]. In addition to their cargo, exosomes also express specific sets of adhesion molecules, integrins, and tetraspanins on their surface, which seem to regulate exosomes uptake by specific target cells by interacting with transmembrane receptors and ligands [35,36,37,38]. Some of these proteins are also involved in exosomes biogenesis [21], a process that will be further described below. 

Exosomes are distinguished from other extracellular vesicles not only because of their small size and distinct composition, but most importantly due to their biogenesis [39]. Exosomes biogenesis begins with the formation of endosomes by endocytosis. This process consists of the inward invagination of the plasma membrane followed by vesicle scission to form early endosomes. When inside the cell, intraluminal vesicles (ILVs) are formed within the lumen of these endosomes through several inward invaginations of their membrane. These mature, ILV-containing endosomes are known as multivesicular bodies (MVBs). The subsequent fusion of MVBs with the cell’s plasma membrane results in the release of the mature ILVs (i.e., exosomes) into the extracellular space [40]. During the MVBs’ journey through the cells cytosol until they fuse with the plasma membrane, specific endosomal proteins and other cellular contents are sorted into ILVs. Both the invagination of the endosomal membrane to form ILVs, and this process of differential sorting of endosomal and cellular contents into exosomes, are mainly controlled by the endosomal sorting complexes required for transport (ESCRT), which include ESCRT-0, ESCRT-I, ESCRT-II, and ESCRT-III [41,42,43]. In fact, studies have shown that the inhibition of ESCRT complex constituents, such as hepatocyte growth factor-regulated tyrosine kinase substrate (HRS) from ESCRT-0 and tumor susceptibility gene 101 (TSG101) from ESCRT-I, can significantly decrease the release of exosomes [44,45,46]. However, exosomes are still formed when the ESCRT-dependent pathway is abrogated, suggesting that the sorting of exosomal cargo is also performed through ESCRT-independent pathways [47]. ESCRT-independent pathways involve lipids, tetraspanins, or heat shock proteins (Hsp) that have been found enriched in exosomes. The sphingolipid ceramide seems to be involved in ILV formation and the blocking of ceramide biogenesis leads to a decrease in exosomes secretion [48]. Cargo sorting is intrinsically linked to tetraspanins, such as CD63 and CD81 (exosomal markers), which have been shown to regulate protein sorting into ILVs [49,50]. Tspan8 was also shown to regulate the sorting of specific proteins and mRNA into exosomes [51]. In maturing reticulocytes, the loading of TFR into exosomes is dependent on its interaction with heat shock cognate 70 (Hsc70) and Alix proteins [52]. Proteins of the Rab family of GTPases have also been implicated in exosomes biogenesis and MVB transport through the cytosol and, more importantly, exosomes release into the extracellular environment. Some examples of Rab proteins involved in these processes are Rab7, Rab11, Rab27a, Rab27b, and Rab35. Rab11 and Rab35 have a closer association with the early stages of ILV formation and cargo sorting, whereas Rab7 and Rab27a/b are associated with late endosomes [43]. Targeting some of these proteins involved in exosomes biogenesis and secretion can be potentially useful for the inhibition of the communication between cancer cells and other cells. The consequent downregulation of cancer exosomes production and release could block the trigger of an immunosuppressive microenvironment by cancer cells via exosomes and the establishment of a metastatic niche on distant organs. The potential use of this strategy for the treatment of cancer is extensively discussed in Bastos et al. [53].

## 3. Cancer Exosomes and Immune Response in PDAC

Over the past years, the spark of interest about exosomes and their roles in cancer has escalated exponentially. As summarized in Table 1, exosomes participate in key processes during tumor progression, including the important step of metastasis. The use of exosomes as nanocarriers of immunotherapeutic agents for the treatment of cancer was proposed based on the increasing evidence of their involvement in the activation or suppression of immune responses in several cancers, including pancreatic cancer (Table 1) [54]. In vitro studies showed that Hsp70-positive pancreatic cancer cells secrete exosomes containing high levels of Hsp70/Bag-4, which enhance the migration and cytolytic activity of natural killer (NK) cells towards Hsp70-positive cancer cells [55]. By contrast, most studies suggest that cancer exosomes actively trigger immunosuppressive responses in PDAC. In PDAC patients, miR-203 is found overexpressed [56]. Upon treatment with miR-203-positive exosomes derived from pancreatic cancer cells, dendritic cells (DCs) downregulate toll-like receptor 4 (TLR4), tumor necrosis factor-α (TNF-α), and interleukin-12 (IL-12) expression [57], which may prevent DC antigen presentation. Loss of TLR4 was shown to abrogate DCs’ capacity to present tumor-specific antigens upon treatment of both mice and humans with breast cancer. In other words, TLR4 expression in DCs is essential for the induction of a sustained immune response against dying tumor cells and for the success of radiotherapy and chemotherapy approaches [58]. Therefore, targeting the release of miR-203-positive exosomes in pancreatic cancer might increase the response of PDAC patients to therapy. MiR-212-3p was also found expressed in pancreatic cancer-derived exosomes, inhibiting the expression of the regulatory factor X-associated protein (RFXAP) and consequently decreasing major histocompatibility complex (MHC) class II expression when released into DCs. As a result, DCs are unable to activate CD4^+^ T cells, potentially contributing to the generation of an immunotolerant microenvironment in PDAC [59]. Downregulation of miR-212-3p in pancreatic cancer cells or inhibition of the secretion of exosomes by cancer cells should be explored as therapeutic strategies to prevent the inhibition of DC antigen-presenting function by miR-212-3p and promote the activation of anti-cancer immune responses. Moreover, Basso et al. showed that conditioned medium from PDAC cells expressing SMAD4 induced an increase in the proliferation of TReg cells while decreasing the CD8^+^ T cell subpopulation [60]. These findings agree with previous studies showing high accumulation of TReg cells and minimal CD8^+^ T cell infiltration in the tumor microenvironment in a PDAC mouse model [13] and patients [61]. In addition, the few CD8^+^ T cells that constitute the tumor immune infiltrate seem to be inactive [13]. Chen et al. [62] offers a possible explanation for the observed impaired infiltration of CD8^+^ T cells. In fact, Chen et al. found high levels of PD-L1-positive exosomes in the circulation of melanoma patients. PD-L1-positive exosomes are secreted by melanoma cells, spread through the circulation, and prevent the proliferation of CD8^+^ T cells as well as their infiltration in the tumor microenvironment. If the same findings apply to PDAC, they might explain the ineffectiveness of anti-PD-L1 immunotherapy and highlight the potential of blocking exosomes secretion and uptake, or even the removal of immunosuppressive exosomes from the circulation as an adjuvant therapy in cancer treatment. A recent study by Maybruck et al. [63] also supports the notion that tumor exosomes induce an immunosuppressive microenvironment because exosomes derived from head and neck cancer cell lines alter CD8^+^ T cells, which adopt a suppressive phenotype and inhibit the proliferation and function of responder T cells. This effect seems to be partially driven by Galectin 1 (Gal-1), which is present in cancer exosomes derived from the Tu167 cell line [63]. In corroboration, the expression of Gal-1 is increased in PDAC mice causing a reduction in T cell infiltration in the tumor microenvironment [64]. Hence, it is possible that, similarly to head and neck cancer cells, PDAC tumor cells load Gal-1 into exosomes, which then results in the suppression of immune responses against the tumor cells. MDSCs and M2 macrophages are also extensively found in the PDAC microenvironment [13,61,65]. The presence of M2 macrophages in the invasive front of pancreatic cancer contributed to tumor progression and lymphangiogenesis, and correlates with lymphatic metastasis [65] and poor survival [61,65,66]. A recent study showed that exosomes derived from pancreatic cancer cell lines cause pro-tumor phenotype changes in macrophages. The induction of this immunosuppressive phenotype in macrophages was more accentuated when macrophages were treated with the ascites-derived, highly metastatic AsPC-1 pancreatic cancer cell line. In addition, macrophages treated with AsPC-1-derived exosomes secrete increased amounts of several growth factors and cytokines which play a key role in the promotion of pancreatic cancer progression, metastasis, and angiogenesis. The secretion of prostaglandin E2 (PGE2) by the treated macrophages was also increased [67]. This is especially relevant as PGE2 can inhibit the maturation of dendritic cells as well as their infiltration in kidney cancer and consequently prevent CD8^+^ T cells activation [68]. PGE2 present on lung cancer cell-conditioned medium has also been shown to induce TReg cells differentiation and activation, thus promoting TReg cells’ suppressive function [69]. If extrapolated to PDAC, these findings mean that the effects of pancreatic cancer exosomes on macrophages could contribute to the establishment of the immunosuppressive microenvironment in PDAC. MDSCs are also heavily present in the pancreatic cancer microenvironment and greatly contribute to the maintenance of an immunosuppressive microenvironment [13,61]. Wang et al. showed that exosomes derived from human pancreatic cancer cells have the capacity to induce differentiation of myeloid cells towards MDSCs in vivo [70]. The mechanisms behind the observed infiltration of MDSCs [13,61] in PDAC are also under study. Incubation of human peripheral blood mononuclear cells with exosomes derived from BxPC3 pancreatic cancer cells leads to an increase in the population of MDSCs and a decrease in DCs. Although these effects seem to be independent of Smad4 expression, exosomes from BxPC3 cells that lack Smad4 prompted more effectively myeloid cells to acquire an immunosuppressive phenotype, which is characterized by increased calcium flux and glycolysis. In fact, BxPC3-Smad4^+^-derived exosomes contain hsa-miR-494-3p and glycolytic enzymes which are transferred into myeloid cells to regulate calcium flux and glycolysis [60].

PDAC is characterized by its high metastatic potential [3], which might be potentiated by cancer exosomes. Due to their ability to enter the circulation, tumor-derived exosomes can travel to distant organs and modulate their environment, turning an otherwise hostile environment, which would hinder tumor cell adhesion and thrive, into a permissive niche. Pancreatic cancer-derived exosomes localize mainly to the lungs and liver, which are markedly regulated by the presence of specific exosomal integrins [71]. Exosomes expressing integrin alpha 6/beta 4 (ITGα6β4) and ITGα6β1 localize to the lungs, where they bind fibroblasts and epithelial cells, whereas exosomes that overexpress ITGα_v_β5 are taken up by Kupffer macrophages in the liver [71]. Exosomes are then able to educate the target cells and trigger inflammatory responses and the recruitment of suppressive immune cells to promote the formation of a pre-metastatic niche [71,72]. In fact, in addition to regulating the recruitment, proliferation, and activation of immune cells within the primary tumor bed, pancreatic cancer-derived exosomes have been shown to play similar roles in distant organs, promoting the recruitment of bone-marrow derived cells and the establishment of a pro-tumorigenic soil in the liver [72,73]. Orthotopic pancreatic cancer models were injected with exosomes derived from Panc02-H7 cells and presented high levels of circulating MDSCs and increased infiltration of myeloid cells in the liver. Panc02-H7 exosomes also triggered the activation of Stat3 in these myeloid infiltrates [73]. This may lead to the activation of the immunosuppressive function of the myeloid cells and aid in the establishment of an immunosuppressive microenvironment that is permissive and even supportive for the development of metastases [74]. Nevertheless, the cascade of events behind immune tolerance at distant sites by pancreatic cancer-derived exosomes remains elusive and should be the subject of further studies.

The vast majority of the currently available data suggests an immunosuppressive role for exosomes in pancreatic cancer. Any discovery related to exosomes function and contents as well as the mechanisms explored by pancreatic cancer cells to induce an immunosuppressive microenvironment via exosomes can be groundbreaking. Efforts should be focused on determining in which way these findings are relevant for cancer therapy, and whether cancer exosomes can be targeted or altered in order to prompt the patient’s own immune system to eradicate cancer cells.

## 4. Advantages of the Use of Exosomes in Immunotherapy

**Figure 1:**
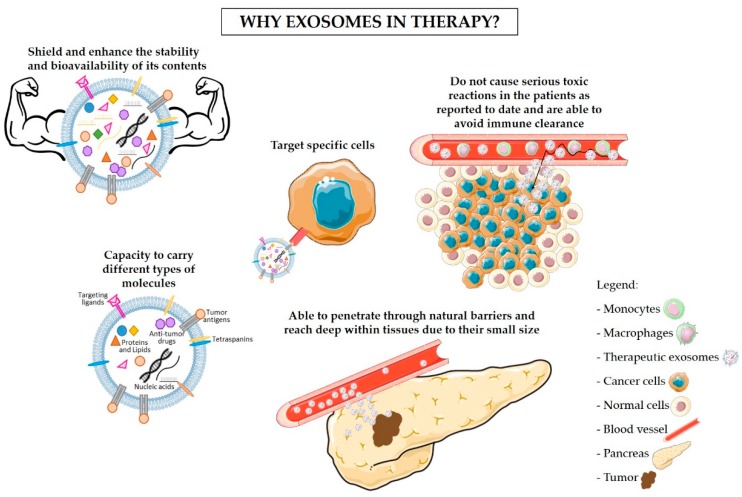
Advantages of using exosomes in therapy. Exosomes are natural carriers of proteins, lipids, and nucleic acids, being crucial for the communication between cells. Therefore, exosomes-based therapy is a promising approach for the treatment of cancer since exosomes can carry and deliver anti-cancer and immunomodulatory drugs, tumor antigens, and siRNA molecules to specifically target cancer cells without causing serious toxic reactions. Exosomes can be produced using the patient’s own cells to prevent the occurrence of an immune reaction to the carriers. Moreover, exosomes act as a shield, protecting their contents from being degraded before reaching the target cells. Their small size also enables them to penetrate through natural barriers and reach deep within tissues.

**Figure 2:**
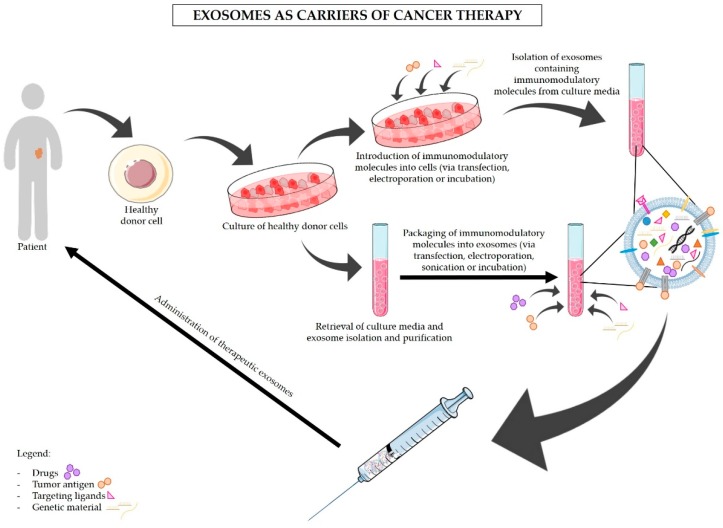
Exosomes as carriers of cancer therapy. Exosomes are potential tools for the delivery of chemotherapy, antigens, and immunotherapy drugs to cancer cells. These exosomes can be isolated from the patient’s own cells, bioengineered to carry specific anti-cancer drugs, tumor-specific antigens, targeting ligands, or genetic material and then administered in the form of exosome-based anti-cancer vaccines. Black arrows are indicative of the work flow for exosomes manipulation and isolation necessary for their use in cancer therapy.

The use of exosomes as nanocarriers of tumor-specific antigens and immunomodulatory drugs is now acknowledged as a promising approach for the treatment of cancer. Due to their endogenous origin and capacity to carry different types of molecules, including proteins, lipids, and RNA, exosomes present multiple advantages in comparison to other nanoparticles [26,87]. The advantages of the use of exosomes in therapy are described below and summarized in Figure 1. One of the most encouraging advantages is the non-toxic and non-immunogenic nature of exosomes. In fact, because of their endogenous origin, we expect that exosomes would not cause serious reactions in the patients, unlike lipid-based nanocarriers (e.g., liposomes) [88,89]. The liposome-associated toxicity is inherent to their synthetic nature and polymer core. Hence, liposomes are recognized as foreign and can trigger an anaphylactic reaction that is characterized by a strong innate immune response [90,91]. In addition, exosomes are naturally secreted by most cells within our organism [92]. By exploring the use of a patient’s own healthy cells for the production of exosomes to be used in cancer treatment (Figure 2), it would be possible to avoid adverse immune reactions to the carriers [93]. Furthermore, exosomes have been reported to avoid phagocytosis by circulating macrophages and monocytes via CD47. Moreover, exosomes show higher efficacy than liposomes in delivering RNAi molecules targeting KRAS^G12D^ to pancreatic cancer cells in vivo, which was in part facilitated by the CD47 protective function [25]. Exosomes also shield and enhance the stability and bioavailability of their contents (specially RNA molecules) that otherwise would be rapidly degraded when passing through the circulation [94,95,96]. On the other hand, exosomes’ small size enables them to penetrate through natural barriers, including the blood–brain barrier [97,98], and reach deep within tissues [99]. Exosomes are taken up by specific target cells, a process that seems to be regulated by the presence of specific adhesion molecules, integrins, and tetraspanins on the exosomes’ surface [35,36,37,38]. For instance, exosomes derived from Tspan8-expressing cells are preferentially taken up by CD54^+^ pancreas and endothelial cells [38]. Hence, future exosomes-based therapy can take advantage of the characteristic to target specific cells. However, this trait also indicates that the use of exosomes as carriers of immunomodulatory drugs should not be taken lightly. Minor variations in exosomal surface proteins can alter exosomes specificity, and a broad knowledge of exosomes biogenesis and of the target cells is necessary for the correct engineering of exosomes [38]. The expression of specific proteins on the surface of exosomes can be controlled by upregulating these proteins in the cells of origin [38,100]. Targeting of the tumor cells can also be improved by anchoring superparamagnetic nanoparticles to exosomes, which are “guided” towards the tumor by moderate magnetic fields [101]. Upon entering the receiving cell, the exosomes’ cargo is released into the cytoplasm, inducing changes in the protein expression [75,102], the activation status of signaling pathways [103], and the cell’s phenotype [104,105,106]. Furthermore, exosomes can either enter into the receiving cell via endocytosis or fuse with the plasma membrane of recipient cells which then incorporates the proteins present in the exosomal membrane [26]. Exosomes are, therefore, a potentially useful tool for the delivery of immunomodulatory agents into specific target cells to block immunosuppression and/or stimulate anti-cancer immune responses. These two approaches could then be used in combination with standard chemotherapy drugs to achieve maximal elimination of pancreatic cancer cells with minimal side effects, as to allow the surgical removal of tumors otherwise inoperable and increase patients’ survival. 

## 5. Exosomes-Based Immunotherapy Approaches for the Treatment of PDAC

**Figure 3:**
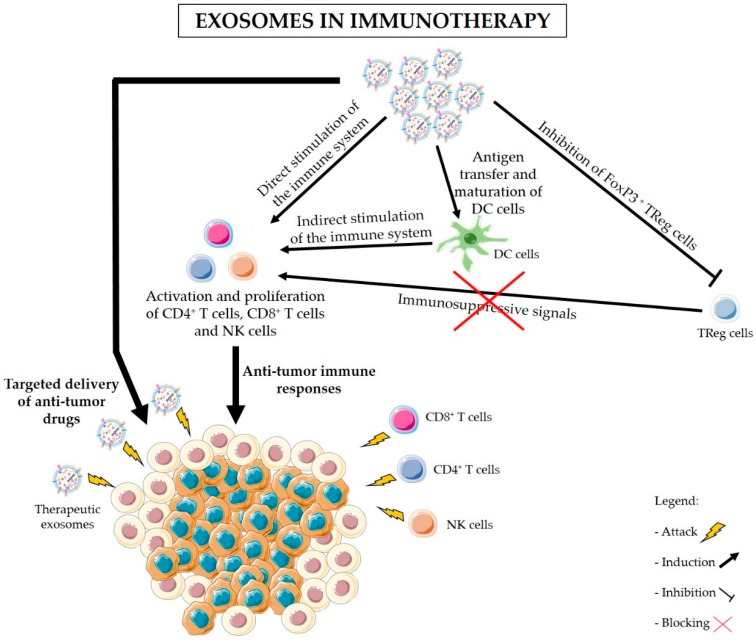
Exosomes in immunotherapy. In addition to exosome-mediated delivery of anti-cancer drugs to target cancer cells, exosomes can be used in immunotherapy to achieve a sustained and specific immune response against cancer cells. Exosomes can present tumor antigens to dendritic cells (DC cells) and promote DC maturation. Mature DCs can then stimulate the activation of cytotoxic T cells and natural killer (NK) cells to kill cancer cells expressing those antigens. T cells and NK cells can also be directly stimulated by exosomes. Nevertheless, tumors possess a characteristic immunosuppressive microenvironment which inhibits the infiltration and activation of T cells or NK cells and, thus, protecting cancer cells against immune attacks. Hence, the use of exosomes-based therapy to inhibit regulatory T cells (TReg cells) and block immunosuppressive signals to T cells also represents a prospective approach in cancer immunotherapy. The combination of immunostimulatory drugs with the inhibition of immunosuppression by TReg cells might be the key for the effective treatment of PDAC.

Various studies have shown a relationship between cancer-derived exosomes and the modulation of immune responses in PDAC. Exosomes are also valuable and effective tools for the delivery of anti-cancer drugs and other molecules, such as RNAi against mutant KRAS, to specifically target PDAC cancer cells (Figure 2) [25,53,107,108]. Figure 3 illustrates the proposed strategies of immunomodulation via exosomes-based immunotherapy for the treatment of pancreatic cancer. In addition, blocking exosomes secretion is also considered a potential strategy for the treatment of cancer [53]. Negligible efforts have been made to fully understand how exosomes can be used to indirectly target pancreatic cancer via stimulation of the immune system. Here we highlight the dual applicability of exosomes in cancer treatment and examine in detail the state-of-the-art work regarding the use of exosomes as carriers of immunotherapy, and the use of exosomes inhibitors to avoid exosomes’ immunosuppressive and tumor-supportive roles. Examples of the uses of exosomes in cancer treatment are also presented in Table 2. Many researchers have dedicated their efforts to find the best strategy to use exosomes as carriers of anti-cancer therapy, and to overcome the current challenges of this delivery system. Exosomes can be obtained from cell cultures or body fluids collected from the patient, modified in vitro according to their final purpose, and then administered to the same patient (Figure 2) [109]. Nevertheless, the main dilemmas associated with therapeutic exosomes are the choice of the cell of origin and the modification method. Stimulation of DCs with cancer peptides for the production of DC-derived exosomes which carry specific antigens is the most well-studied option. These exosomes share DCs’ ability to present specific antigens to T cells and to promote an immune response against the patients’ cancer cells (Figure 2 and Figure 3) [27]. In fact, exosomes secreted by DCs contain MHC class I and II on their surface as well as other costimulatory molecules that promote the activation of effector T cells and NK cells. It is believed that exosomes can stimulate cytotoxic immune cells via direct MHC-mediated antigen presentation [110,111]. Similar effects seem to be driven by B cell-derived exosomes, highlighting their usefulness for cancer immunotherapy [112]. Even though with limited efficacy, the immune stimulatory effects of DC-derived exosomes have been confirmed in advanced non-small lung cancer patients that have been pre-treated with chemotherapy. This phase II clinical trial tested the use of DC-derived exosomes as immunotherapy. Treatment with DC-derived exosomes proved to be effective in stimulating NK cell activation and improved the patient’s progression-free survival but only in a subset of patients [113]. This study is a clear indicator of the suitability of using exosomes derived from antigen-presenting cells to boost immune responses against cancer; however, it also reminds us that extensive work in optimizing and standardizing protocols must be carried out before this therapeutic strategy can be used in a clinical context. Tumor-associated ascites also contain high amounts of exosomes that, when administered together with granulocyte-macrophage colony-stimulating factor (GM-CSF), have been found to stimulate T cell-driven responses against colorectal cancer cells [114]. Whether PDAC-associated ascites exosomes can have the same beneficial effects remains to be seen. However, triggering T cell responses to kill cancer cells might not be enough (Figure 3). In PDAC, the immune infiltrate is mostly constituted by TReg cells [13,61]. As a result, PDAC cancer cells are surrounded by an overwhelming immunosuppressive microenvironment that protects them against immune attacks. Thus, it is important to find how useful exosomes can be in the inhibition of TReg cells’ suppressive function. Cancer exosomes have been reported to stimulate TReg cells’ suppressive role by influencing gene expression in TReg cells [115]. Exosomes derived from different tumor cell lines and that carry transforming growth factor β1 (TGF-β1) and interleukin 10 (IL-10) were able to promote CD4^+^CD25^−^ T cell differentiation into TReg cells. These tumor-derived exosomes also promoted TReg cells’ proliferation and immunosuppressive function [116]. Further investigation on the signals and mechanisms involved in the activation of TReg cells by tumor exosomes, specifically in the context of PDAC, can lead to the discovery of therapeutic alternatives for the regulation of TReg cell function (Figure 2 and Figure 3).

Research about the use of exosomes in PDAC immunotherapy is still scarce. Nevertheless, a recent in vivo study has shown that the combination of chemotherapy drugs commonly used for PDAC treatment (i.e., all-trans retinoic acid - ATRA, sunitinib, and gemcitabine) with vaccines containing DCs loaded with pancreatic cancer-derived exosomes, efficiently inhibits the growth of metastases and prolonged mice survival [117]. It was shown that these chemotherapeutic drugs alone could impair MDSC infiltration and activation. However, a higher recruitment and activation of effector T cells and better prognosis were obtained when ATRA, sunitinib, or gemcitabine was administered together with tumor exosomes-loaded DCs [117]. Targeting macrophages is also a promising approach for the treatment of pancreatic cancer. Su et al. showed that exosomes derived from Panc-1 cells reprogram M1 macrophages to adopt an M2 phenotype. However, this could be reversed by treating the converted M2 macrophages with exosomes from Panc-1 cells that were transfected with miR-155 and miR-125b2. As a result, the M2 macrophages were reprogrammed back to an M1, anti-tumor phenotype [118]. Therefore, bioengineering tumor exosomes to express specific miRNAs that can cause phenotypic changes in macrophages and inhibit their immunosuppressive and pro-tumor activity should be further explored. On the other hand, another study shows that macrophage-derived exosomes caused resistance to gemcitabine in mice that were injected with PDAC cells derived from a KPC mouse. In the same study, the authors show that mice deficient in Rab27a and Rab27b, treated with gemcitabine, exhibited smaller tumors than wild-type mice [81]. Even though the focus of this study was not the development of exosomes-based immunotherapy, this study shows that the exosomes-mediated communication between pancreatic cancer cells and immune cells contributes to pancreatic cancer’s poor prognosis and resistance to therapy. It also suggests that the combination of chemotherapeutic drugs with inhibitors of Rab27a/b or other inhibitors of exosomes secretion could lead to significant improvements in a patient’s response to therapy and survival. Therefore, in addition to the use of exosomes as nanocarriers of immunomodulatory agents, it is also possible to trigger anti-cancer immune responses by inhibiting the secretion of cancer-derived exosomes and, consequently, hinder cancer cells’ ability to reprogram their microenvironment and block the infiltration and activation of cytotoxic T cells. An increasing number of studies shows that the inhibition of exosomes secretion can impair tumor growth and metastasis in pancreatic cancer [80] as well as other tumors [119,120]. The combination of gemcitabine with the exosomes inhibitor GW4869 could also prevent chemoresistance [80]. However, this approach entails the inhibition of exosomes secretion by both cancer cells and normal cells, and its use for the treatment of cancer should be carefully considered as exosomes play crucial roles in the regulation of normal biological functions. Thus, the general inhibition of exosomes secretion could have catastrophic consequences. A safer approach would be to inhibit specific proteins that are known to carry out the pro-tumorigenic and pro-metastatic functions of exosomes. For example, Hoshino et al. found that the knockdown or inhibition of ITGβ4 or ITGβ5, which are found overexpressed in pancreatic cancer-derived exosomes, was sufficient to significantly decrease the capacity of pancreatic cancer cells to form metastases in the lungs and liver, respectively [71]. On the other hand, if the specific targeting of cancer-derived exosomes becomes a reality, a new set of therapeutic possibilities would arise with the potential to revolutionize the way we treat cancer. Recently, the first step in identifying compounds that are able to inhibit the secretion of exosomes by cancer cells was taken by Datta et al., which performed a quantitative high throughput screen (qHTS) of two drug libraries and were able to identify several compounds with the capacity to modulate exosomes biogenesis and secretion by C4-2B prostate cancer cells [121].

To our knowledge, no other studies have been conducted using exosomes to deliver immunotherapy drugs, antigens, cytokines, or any other immunostimulatory molecules, in PDAC. We believe that exosomes-based immunotherapy is a very promising approach for the treatment of this disease that should be carefully studied. The inhibition of exosomes secretion has also been proven efficient for the treatment of cancer [53,80]. Nevertheless, both of these applications present challenges, mostly related to the production of exosomes on a large scale, isolation methods, therapeutic cargo loading, and dosage [53,122], as well as the possible side effects derived from blocking exosomes secretion from healthy cells [123]. Additionally, the best cell source to obtain exosomes for therapeutic purposes is still debatable. We advocate the use of healthy cells isolated from the patients themselves to obtain exosomes, which can then be engineered to carry immunomodulatory molecules to specific cells. As a result, these vesicles are able to evade the immune system and safely travel through the blood stream to activate specific immune responses against cancer cells. With this review, we hope to set the stage to a better understanding of the benefits and risks of therapeutic approaches using exosomes, and finding new ways to overcome the challenges that these approaches currently face.

## 6. Final Remarks 

Exosomes are small vesicles of endogenous origin that mediate the communication between neighbor or even distant cells [26]. Due to their stability [25,93] and ability to overcome natural barriers [97,98], exosomes from different origins can be found in circulation and target specific cells within distant tissues [38,99]. These characteristics are what makes exosomes a suitable vehicle for cancer chemotherapy and immunotherapy drugs. Moreover, exosomes have been previously shown to participate in the stimulation of immune responses against cancer cells [55] as well as in the establishment of an immunosuppressive environment [57,60] commonly found associated with PDAC [13,61]. Over the past few years, one of the therapeutic approaches frequently suggested for the treatment of PDAC patients combines the use of chemotherapy with inhibitors of immunosuppression and/or agents that stimulate immune cytotoxic responses against cancer cells [12,14,20]. The use of exosomes in PDAC immunotherapy can mean great improvements in the survival and quality of life of PDAC patients. Currently, only two reports have focused on developing and testing exosomes-based delivery of immunomodulatory agents to tumors. One consisted of a preclinical study and focused on the administration of vaccines containing DCs that enclosed tumor-derived exosomes in order to induce T cell-driven anti-tumor immune responses. Such an approach could be combined with current chemotherapeutic drugs to achieve greater cancer cell death [117]. The other showed in vitro that exosomes derived from Panc-1 cells expressing miR-155 and miR-125b2 could reprogram M2 macrophages back to an M1 phenotype, which could then impair tumor growth [118]. There is still a horizon of possibilities to be explored when we consider the use of exosomes-based immunotherapies for PDAC treatment. To conclude, one can foresee that the use of exosomes in a clinical setting can revolutionize the treatment of cancer and other pathologies. It is therefore imperative to further study the role of exosomes in cancer and immune modulation and to use this knowledge for the development of new exosomes-based therapies. 

## Figures and Tables

**Table 1:** Multiple roles of exosomes in cancer.

Function in Cancer	Specific Role	References
**Help Tumor Cells to Evade the Immune System**	Activate NK cells	[55]
	Prevent DC antigen presentation	[57,59]
	Favor an immunosuppressive phenotype in macrophages	[67]
	Induce differentiation of myeloid cells towards MDSCs	[70]
	Induce proliferation of TReg cells	[60]
	Prevent proliferation and activation of effector T cells	[62,64]
**Drive Tumor Development and Progression**	Induce tumor growth and transformation and inhibit cancer cell death	[75,76,77,78]
	Stimulate angiogenesis	[51,79]
	Contribute to drug resistance	[80,81]
	Extracellular matrix remodeling	[82,83]
	Promote EMT	[84,85]
	Induce metabolic reprograming	[86]
**Promote metastasis**	Educate the cells and trigger inflammatory responses at the metastatic site	[71,72]
	Promote the recruitment of suppressive immune cells to promote the formation of a pre-metastatic niche	[71,72,73,74]

**Abbreviations:** dendritic cells, DCs; epithelial–mesenchymal transition, EMT; myeloid-derived suppressor cells, MDSCs; natural killer cells, NK cells; regulatory T cells, TReg cells.

**Table 2:** Examples of the possible uses of exosomes for the treatment of cancer.

Treatment Options		Cell of Origin	Type of Modification/Effect	Research	Main Findings	Ref
**Delivery of immuno-stimulatory molecules**	**Small molecules**	Reticulocytes	Incubation of exosomal solution with doxorubicin	Delivery of doxorubicin to tumor cells via exosomes anchored to superparamagnetic nanoparticles	Doxorubicin was more effectively delivered to tumor cells via exosomes which were anchored to superparamagnetic nanoparticles; mice inoculated subcutaneously with H22 hepatocarcinoma cells presented decreased tumor growth when treated with these exosomes.	[101]
		Murine macrophages (i.e., RAW 264.7 cell line)	Sonication to load paclitaxel into exosomes	Study the efficacy of paclitaxel for the treatment of multiple drug resistant cancers when delivered via exosomes	Exosomes-delivered paclitaxel successfully reached tumor cells and inhibited the growth of pulmonary metastases in vivo (in mice injected via intra-tail vein with 3LL-M27 cells).	[124]
	**Antibodies and antigens**	Monocyte-derived, IFNγ-maturated DCs	Passive intracellular sorting of MHC molecules	Presentation of cancer antigens to cytotoxic immune cells via MHC-I and MHC-II expressed on the surface of DC-derived exosomes	Exosomes derived from IFNγ-maturated DCs stimulate the activation of NK cells, but not T cells; such effect is sufficient to improve the rate of progression-free survival in unresectable non-small cell lung cancer patients. However, these DC-derived exosomes were only effective as maintenance immunotherapy in less than 50% of the patient cohort.	[113]
		Monocyte-derived, immature DCs	Pulsing of exosomal MHC-I proteins with Mart1 via acid elution	Presentation of cancer antigens to cytotoxic immune cells via MHC-I expressed on the surface of DC-derived exosomes	Exosomes containing MHC-I/Mart1 complexes are able to activate CD8^+^ T cells.	[125]
		Mouse bone marrow-derived DCs	Treatment of DCs with exosomes isolated from UNKC6141 murine pancreatic cancer cells	Study the activation of immune responses and survival in pancreatic cancer orthotopic mice models treated with tumor exosomes-loaded DCs	Tumor exosomes-loaded DCs were able to activate and recruit effector T cells to pancreatic cancer and improve prognosis when administered together with ATRA, sunitinib, or gemcitabine.	[117]
		HLA-DR15-positive human B cells	Incubation of cells and exosomes with Hsp65 antigen or antigenic peptide.	Study whether B cell-derived exosomes are able to activate T cells via MHC-mediated presentation of Hsp65 antigen	Exosomes incubated with Hsp65 or derived from the medium of B cells pre-incubated with Hsp65 were able to activate T cells.	[112]
	**siRNA and RNAi**	Human plasma cells	Electroporation of siRNA	Delivery of siRNA against MAPK1 to monocytes and T cells	Electroporated exosomes are able to enter human monocytes and lymphocytes (in vitro), deliver siRNA and successfully downregulate MAPK-1 transcription.	[109]
		Human foreskin fibroblast cell line (i.e., BJ cell line)	Electroporation of RNAi molecules	Delivery of RNAi molecules against the mutant KRAS^G12D^ to pancreatic cancer cells	BJ-derived exosomes expressing RNAi against KRAS^G12D^ are able to specifically target mouse pancreatic cancer cells in vivo, diminishing the expression of KRAS^G12D^ and considerably diminishing cancer proliferation and metastasis, which resulted in an increase in mice overall survival.	[25]
	**miRNA**	Human pancreatic ductal adenocarcinoma cell line Panc-1	Transfection of DNA plasmids for miR-155 and miR-125b2 into Panc-1 cells	Study phenotype changes on J774A.1 murine macrophages when treated with exosomes that overexpress miR-155 and miR-125b2	M2 macrophages treated with exosomes derived from the transfected Panc-1 cells were reprogrammed back to an M1, anti-tumor phenotype.	[118]
**Blocking exosomes biogenesis and secretion**	**GW4869**	All cells	Inhibition of exosomes biogenesis	Study the combination of gemcitabine with an inhibitor of exosomes biogenesis as a treatment option	GW4869 prevented chemoresistance caused by the increased amount of exosomes released by CAFs that were exposed to gemcitabine.	[80]
	**Inhibition of Rab27a**	4T1 murine breast cancer cells	Infection with shRNA-expressing lentiviruses	Study of the role of Rab27a in exosomes secretion as well as in breast cancer growth and metastasis.	Blockade of Rab27a and, thus, of exosomes secretion decreased tumor growth and lung metastasis in mice inoculated with metastatic 4T1 cells.	[119]

Abbreviations: all-trans retinoic acid, ATRA; cancer-associated fibroblasts, CAFs; dendritic cells, DC; heat shock protein 65, Hsp65; interferon, IFN; major histocompatibility complex, MHC; mitogen-activated protein kinase 1, MAPK-1; natural killer, NK.

## References

[1] Ferlay J., Ervik M., Lam F., Colombet M., Mery L., Piñeros M., Znaor A., Soerjomataram I., Bray F. (2018). Global Cancer Observatory. Lyon, France: International Agency for Research on Cancer. https://gco.Iarc.Fr.

[2] Bray F., Ferlay J., Soerjomataram I., Siegel R.L., Torre L.A., Jemal A. (2018). Global cancer statistics 2018: Globocan estimates of incidence and mortality worldwide for 36 cancers in 185 countries. CA Cancer J. Clin..

[3] Hidalgo M., Cascinu S., Kleeff J., Labianca R., Löhr J.M., Neoptolemos J., Real F.X., Van Laethem J.L., Heinemann V. (2015). Addressing the challenges of pancreatic cancer: Future directions for improving outcomes. Pancreatology.

[4] Huang L., Jansen L., Balavarca Y., Babaei M., van der Geest L., Lemmens V., Van Eycken L., De Schutter H., Johannesen T.B., Primic-Žakelj M. (2018). Stratified survival of resected and overall pancreatic cancer patients in europe and the USA in the early twenty-first century: A large, international population-based study. BMC Med..

[5] Ilic M., Ilic I. (2016). Epidemiology of pancreatic cancer. World J. Gastroenterol..

[6] Siegel R.L., Miller K.D., Jemal A. (2018). Cancer statistics, 2018. CA Cancer J. Clin..

[7] Garrido-Laguna I., Hidalgo M. (2015). Pancreatic cancer: From state-of-the-art treatments to promising novel therapies. Nat. Rev. Clin. Oncol..

[8] Werner J., Combs S.E., Springfeld C., Hartwig W., Hackert T., Büchler M.W. (2013). Advanced-stage pancreatic cancer: Therapy options. Nat. Rev. Clin. Oncol..

[9] Groot V.P., Rezaee N., Wu W., Cameron J.L., Fishman E.K., Hruban R.H., Weiss M.J., Zheng L., Wolfgang C.L., He J. (2018). Patterns, timing, and predictors of recurrence following pancreatectomy for pancreatic ductal adenocarcinoma. Ann. Surg..

[10] Karachristos A., Esnaola N.F. (2014). Surgical management of pancreatic neoplasms: What’s new?. Curr. Gastroenterol. Rep..

[11] Artinyan A., Anaya D.A., McKenzie S., Ellenhorn J.D., Kim J. (2011). Neoadjuvant therapy is associated with improved survival in resectable pancreatic adenocarcinoma. Cancer.

[12] Inman K.S., Francis A.A., Murray N.R. (2014). Complex role for the immune system in initiation and progression of pancreatic cancer. World J. Gastroenterol..

[13] Clark C.E., Hingorani S.R., Mick R., Combs C., Tuveson D.A., Vonderheide R.H. (2007). Dynamics of the immune reaction to pancreatic cancer from inception to invasion. Cancer Res..

[14] Sahin I.H., Askan G., Hu Z.I., O’Reilly E.M. (2017). Immunotherapy in pancreatic ductal adenocarcinoma: An emerging entity?. Ann. Oncol..

[15] Winograd R., Byrne K.T., Evans R.A., Odorizzi P.M., Meyer A.R., Bajor D.L., Clendenin C., Stanger B.Z., Furth E.E., Wherry E.J. (2015). Induction of t-cell immunity overcomes complete resistance to pd-1 and ctla-4 blockade and improves survival in pancreatic carcinoma. Cancer Immunol. Res..

[16] Royal R.E., Levy C., Turner K., Mathur A., Hughes M., Kammula U.S., Sherry R.M., Topalian S.L., Yang J.C., Lowy I. (2010). Phase 2 trial of single agent ipilimumab (anti-ctla-4) for locally advanced or metastatic pancreatic adenocarcinoma. J. Immunother..

[17] Aglietta M., Barone C., Sawyer M.B., Moore M.J., Miller W.H., Bagalà C., Colombi F., Cagnazzo C., Gioeni L., Wang E. (2014). A phase i dose escalation trial of tremelimumab (cp-675,206) in combination with gemcitabine in chemotherapy-naive patients with metastatic pancreatic cancer. Ann. Oncol..

[18] Brahmer J.R., Tykodi S.S., Chow L.Q., Hwu W.J., Topalian S.L., Hwu P., Drake C.G., Camacho L.H., Kauh J., Odunsi K. (2012). Safety and activity of anti-pd-l1 antibody in patients with advanced cancer. N. Engl. J. Med..

[19] Topalian S.L., Drake C.G., Pardoll D.M. (2015). Immune checkpoint blockade: A common denominator approach to cancer therapy. Cancer Cell.

[20] Guo S., Contratto M., Miller G., Leichman L., Wu J. (2017). Immunotherapy in pancreatic cancer: Unleash its potential through novel combinations. World J. Clin. Oncol..

[21] Théry C., Zitvogel L., Amigorena S. (2002). Exosomes: Composition, biogenesis and function. Nat. Rev. Immunol..

[22] Tkach M., Théry C. (2016). Communication by extracellular vesicles: Where we are and where we need to go. Cell.

[23] Kalluri R. (2016). The biology and function of exosomes in cancer. J. Clin. Investig..

[24] Kalluri R., LeBleu V.S. (2016). Discovery of double-stranded genomic DNA in circulating exosomes. Cold Spring Harb. Symp. Quant. Biol..

[25] Kamerkar S., LeBleu V.S., Sugimoto H., Yang S., Ruivo C.F., Melo S.A., Lee J.J., Kalluri R. (2017). Exosomes facilitate therapeutic targeting of oncogenic kras in pancreatic cancer. Nature.

[26] Théry C., Ostrowski M., Segura E. (2009). Membrane vesicles as conveyors of immune responses. Nat. Rev. Immunol..

[27] Pitt J.M., Charrier M., Viaud S., André F., Besse B., Chaput N., Zitvogel L. (2014). Dendritic cell-derived exosomes as immunotherapies in the fight against cancer. J. Immunol..

[28] Pan B.T., Johnstone R.M. (1983). Fate of the transferrin receptor during maturation of sheep reticulocytes in vitro: Selective externalization of the receptor. Cell.

[29] Rajagopal C., Harikumar K.B. (2018). The origin and functions of exosomes in cancer. Front. Oncol..

[30] EL Andaloussi S., Mäger I., Breakefield X.O., Wood M.J. (2013). Extracellular vesicles: Biology and emerging therapeutic opportunities. Nat. Rev. Drug Discov..

[31] Thakur B.K., Zhang H., Becker A., Matei I., Huang Y., Costa-Silva B., Zheng Y., Hoshino A., Brazier H., Xiang J. (2014). Double-stranded DNA in exosomes: A novel biomarker in cancer detection. Cell. Res..

[32] Yang S., Che S.P., Kurywchak P., Tavormina J.L., Gansmo L.B., Correa de Sampaio P., Tachezy M., Bockhorn M., Gebauer F., Haltom A.R. (2017). Detection of mutant kras and tp53 DNA in circulating exosomes from healthy individuals and patients with pancreatic cancer. Cancer Biol. Ther..

[33] Zhang X., Yuan X., Shi H., Wu L., Qian H., Xu W. (2015). Exosomes in cancer: Small particle, big player. J. Hematol. Oncol..

[34] Taylor D.D., Gercel-Taylor C. (2008). Microrna signatures of tumor-derived exosomes as diagnostic biomarkers of ovarian cancer. Gynecol. Oncol..

[35] Morelli A.E., Larregina A.T., Shufesky W.J., Sullivan M.L., Stolz D.B., Papworth G.D., Zahorchak A.F., Logar A.J., Wang Z., Watkins S.C. (2004). Endocytosis, intracellular sorting, and processing of exosomes by dendritic cells. Blood.

[36] Segura E., Guérin C., Hogg N., Amigorena S., Théry C. (2007). Cd8+ dendritic cells use lfa-1 to capture mhc-peptide complexes from exosomes in vivo. J. Immunol..

[37] Nolte-’t Hoen E.N., Buschow S.I., Anderton S.M., Stoorvogel W., Wauben M.H. (2009). Activated t cells recruit exosomes secreted by dendritic cells via lfa-1. Blood.

[38] Rana S., Yue S., Stadel D., Zöller M. (2012). Toward tailored exosomes: The exosomal tetraspanin web contributes to target cell selection. Int. J. Biochem. Cell Biol..

[39] Minciacchi V.R., Freeman M.R., Di Vizio D. (2015). Extracellular vesicles in cancer: Exosomes, microvesicles and the emerging role of large oncosomes. Semin. Cell Dev. Biol..

[40] Cordonnier M., Chanteloup G., Isambert N., Seigneuric R., Fumoleau P., Garrido C., Gobbo J. (2017). Exosomes in cancer theranostic: Diamonds in the rough. Cell. Adh. Migr..

[41] Hurley J.H. (2008). Escrt complexes and the biogenesis of multivesicular bodies. Curr. Opin. Cell Biol..

[42] Williams R.L., Urbé S. (2007). The emerging shape of the escrt machinery. Nat. Rev. Mol. Cell Biol..

[43] Colombo M., Raposo G., Théry C. (2014). Biogenesis, secretion, and intercellular interactions of exosomes and other extracellular vesicles. Annu. Rev. Cell Dev. Biol..

[44] Tamai K., Tanaka N., Nakano T., Kakazu E., Kondo Y., Inoue J., Shiina M., Fukushima K., Hoshino T., Sano K. (2010). Exosome secretion of dendritic cells is regulated by hrs, an escrt-0 protein. Biochem. Biophys. Res. Commun..

[45] Gross J.C., Chaudhary V., Bartscherer K., Boutros M. (2012). Active wnt proteins are secreted on exosomes. Nat. Cell Biol..

[46] Colombo M., Moita C., van Niel G., Kowal J., Vigneron J., Benaroch P., Manel N., Moita L.F., Théry C., Raposo G. (2013). Analysis of escrt functions in exosome biogenesis, composition and secretion highlights the heterogeneity of extracellular vesicles. J. Cell Sci..

[47] Stuffers S., Sem Wegner C., Stenmark H., Brech A. (2009). Multivesicular endosome biogenesis in the absence of escrts. Traffic.

[48] Trajkovic K., Hsu C., Chiantia S., Rajendran L., Wenzel D., Wieland F., Schwille P., Brügger B., Simons M. (2008). Ceramide triggers budding of exosome vesicles into multivesicular endosomes. Science.

[49] Van Niel G., Charrin S., Simoes S., Romao M., Rochin L., Saftig P., Marks M.S., Rubinstein E., Raposo G. (2011). The tetraspanin cd63 regulates escrt-independent and -dependent endosomal sorting during melanogenesis. Dev. Cell.

[50] Perez-Hernandez D., Gutiérrez-Vázquez C., Jorge I., López-Martín S., Ursa A., Sánchez-Madrid F., Vázquez J., Yáñez-Mó M. (2013). The intracellular interactome of tetraspanin-enriched microdomains reveals their function as sorting machineries toward exosomes. J. Biol. Chem..

[51] Nazarenko I., Rana S., Baumann A., McAlear J., Hellwig A., Trendelenburg M., Lochnit G., Preissner K.T., Zöller M. (2010). Cell surface tetraspanin tspan8 contributes to molecular pathways of exosome-induced endothelial cell activation. Cancer Res..

[52] Géminard C., De Gassart A., Blanc L., Vidal M. (2004). Degradation of ap2 during reticulocyte maturation enhances binding of hsc70 and alix to a common site on tfr for sorting into exosomes. Traffic.

[53] Bastos N., Ruivo C.F., da Silva S., Melo S.A. (2018). Exosomes in cancer: Use them or target them?. Semin. Cell. Dev. Biol..

[54] Ruivo C.F., Adem B., Silva M., Melo S.A. (2017). The biology of cancer exosomes: Insights and new perspectives. Cancer Res..

[55] Gastpar R., Gehrmann M., Bausero M.A., Asea A., Gross C., Schroeder J.A., Multhoff G. (2005). Heat shock protein 70 surface-positive tumor exosomes stimulate migratory and cytolytic activity of natural killer cells. Cancer Res..

[56] Bloomston M., Frankel W.L., Petrocca F., Volinia S., Alder H., Hagan J.P., Liu C.G., Bhatt D., Taccioli C., Croce C.M. (2007). Microrna expression patterns to differentiate pancreatic adenocarcinoma from normal pancreas and chronic pancreatitis. JAMA.

[57] Zhou M., Chen J., Zhou L., Chen W., Ding G., Cao L. (2014). Pancreatic cancer derived exosomes regulate the expression of tlr4 in dendritic cells via mir-203. Cell. Immunol..

[58] Apetoh L., Ghiringhelli F., Tesniere A., Obeid M., Ortiz C., Criollo A., Mignot G., Maiuri M.C., Ullrich E., Saulnier P. (2007). Toll-like receptor 4-dependent contribution of the immune system to anticancer chemotherapy and radiotherapy. Nat. Med..

[59] Ding G., Zhou L., Qian Y., Fu M., Chen J., Xiang J., Wu Z., Jiang G., Cao L. (2015). Pancreatic cancer-derived exosomes transfer mirnas to dendritic cells and inhibit rfxap expression via mir-212-3p. Oncotarget.

[60] Basso D., Gnatta E., Padoan A., Fogar P., Furlanello S., Aita A., Bozzato D., Zambon C.F., Arrigoni G., Frasson C. (2017). Pdac-derived exosomes enrich the microenvironment in mdscs in a smad4-dependent manner through a new calcium related axis. Oncotarget.

[61] Ino Y., Yamazaki-Itoh R., Shimada K., Iwasaki M., Kosuge T., Kanai Y., Hiraoka N. (2013). Immune cell infiltration as an indicator of the immune microenvironment of pancreatic cancer. Br. J. Cancer.

[62] Chen G., Huang A.C., Zhang W., Zhang G., Wu M., Xu W., Yu Z., Yang J., Wang B., Sun H. (2018). Exosomal pd-l1 contributes to immunosuppression and is associated with anti-pd-1 response. Nature.

[63] Maybruck B.T., Pfannenstiel L.W., Diaz-Montero M., Gastman B.R. (2017). Tumor-derived exosomes induce cd8+ t cell suppressors. J. Immunother. Cancer.

[64] Martínez-Bosch N., Fernández-Barrena M.G., Moreno M., Ortiz-Zapater E., Munné-Collado J., Iglesias M., André S., Gabius H.J., Hwang R.F., Poirier F. (2014). Galectin-1 drives pancreatic carcinogenesis through stroma remodeling and hedgehog signaling activation. Cancer Res..

[65] Kurahara H., Shinchi H., Mataki Y., Maemura K., Noma H., Kubo F., Sakoda M., Ueno S., Natsugoe S., Takao S. (2011). Significance of m2-polarized tumor-associated macrophage in pancreatic cancer. J. Surg. Res..

[66] Hu H., Hang J.J., Han T., Zhuo M., Jiao F., Wang L.W. (2016). The m2 phenotype of tumor-associated macrophages in the stroma confers a poor prognosis in pancreatic cancer. Tumour. Biol..

[67] Linton S.S., Abraham T., Liao J., Clawson G.A., Butler P.J., Fox T., Kester M., Matters G.L. (2018). Tumor-promoting effects of pancreatic cancer cell exosomes on thp-1-derived macrophages. PLoS ONE.

[68] Ahmadi M., Emery D.C., Morgan D.J. (2008). Prevention of both direct and cross-priming of antitumor cd8+ t-cell responses following overproduction of prostaglandin e2 by tumor cells in vivo. Cancer Res..

[69] Baratelli F., Lin Y., Zhu L., Yang S.C., Heuzé-Vourc’h N., Zeng G., Reckamp K., Dohadwala M., Sharma S., Dubinett S.M. (2005). Prostaglandin e2 induces foxp3 gene expression and t regulatory cell function in human cd4+ t cells. J. Immunol..

[70] Wang Z., von Au A., Schnölzer M., Hackert T., Zöller M. (2016). Cd44v6-competent tumor exosomes promote motility, invasion and cancer-initiating cell marker expression in pancreatic and colorectal cancer cells. Oncotarget.

[71] Hoshino A., Costa-Silva B., Shen T.L., Rodrigues G., Hashimoto A., Tesic Mark M., Molina H., Kohsaka S., Di Giannatale A., Ceder S. (2015). Tumour exosome integrins determine organotropic metastasis. Nature.

[72] Costa-Silva B., Aiello N.M., Ocean A.J., Singh S., Zhang H., Thakur B.K., Becker A., Hoshino A., Mark M.T., Molina H. (2015). Pancreatic cancer exosomes initiate pre-metastatic niche formation in the liver. Nat. Cell Biol..

[73] Yu Z., Zhao S., Ren L., Wang L., Chen Z., Hoffman R.M., Zhou J. (2017). Pancreatic cancer-derived exosomes promote tumor metastasis and liver pre-metastatic niche formation. Oncotarget.

[74] Chalmin F., Ladoire S., Mignot G., Vincent J., Bruchard M., Remy-Martin J.P., Boireau W., Rouleau A., Simon B., Lanneau D. (2010). Membrane-associated hsp72 from tumor-derived exosomes mediates stat3-dependent immunosuppressive function of mouse and human myeloid-derived suppressor cells. J. Clin. Investig..

[75] Melo S.A., Sugimoto H., O’Connell J.T., Kato N., Villanueva A., Vidal A., Qiu L., Vitkin E., Perelman L.T., Melo C.A. (2014). Cancer exosomes perform cell-independent microrna biogenesis and promote tumorigenesis. Cancer Cell..

[76] Abd Elmageed Z.Y., Yang Y., Thomas R., Ranjan M., Mondal D., Moroz K., Fang Z., Rezk B.M., Moparty K., Sikka S.C. (2014). Neoplastic reprogramming of patient-derived adipose stem cells by prostate cancer cell-associated exosomes. Stem Cells.

[77] Takikawa T., Masamune A., Yoshida N., Hamada S., Kogure T., Shimosegawa T. (2017). Exosomes derived from pancreatic stellate cells: Microrna signature and effects on pancreatic cancer cells. Pancreas.

[78] Beloribi-Djefaflia S., Siret C., Lombardo D. (2015). Exosomal lipids induce human pancreatic tumoral miapaca-2 cells resistance through the cxcr4-sdf-1α signaling axis. Oncoscience.

[79] Kucharzewska P., Christianson H.C., Welch J.E., Svensson K.J., Fredlund E., Ringnér M., Mörgelin M., Bourseau-Guilmain E., Bengzon J., Belting M. (2013). Exosomes reflect the hypoxic status of glioma cells and mediate hypoxia-dependent activation of vascular cells during tumor development. Proc. Natl. Acad. Sci. USA.

[80] Richards K.E., Zeleniak A.E., Fishel M.L., Wu J., Littlepage L.E., Hill R. (2017). Cancer-associated fibroblast exosomes regulate survival and proliferation of pancreatic cancer cells. Oncogene.

[81] Binenbaum Y., Fridman E., Yaari Z., Milman N., Schroeder A., Ben David G., Shlomi T., Gil Z. (2018). Transfer of mirna in macrophage-derived exosomes induces drug resistance in pancreatic adenocarcinoma. Cancer Res..

[82] Hendrix A., Maynard D., Pauwels P., Braems G., Denys H., Van den Broecke R., Lambert J., Van Belle S., Cocquyt V., Gespach C. (2010). Effect of the secretory small gtpase rab27b on breast cancer growth, invasion, and metastasis. J. Natl. Cancer Inst..

[83] Sidhu S.S., Mengistab A.T., Tauscher A.N., LaVail J., Basbaum C. (2004). The microvesicle as a vehicle for emmprin in tumor-stromal interactions. Oncogene.

[84] Li Z., Jiang P., Li J., Peng M., Zhao X., Zhang X., Chen K., Zhang Y., Liu H., Gan L. (2018). Tumor-derived exosomal lnc-sox2ot promotes emt and stemness by acting as a cerna in pancreatic ductal adenocarcinoma. Oncogene.

[85] Blackwell R.H., Foreman K.E., Gupta G.N. (2017). The role of cancer-derived exosomes in tumorigenicity & epithelial-to-mesenchymal transition. Cancers (Basel).

[86] Wang L., Zhang B., Zheng W., Kang M., Chen Q., Qin W., Li C., Zhang Y., Shao Y., Wu Y. (2017). Exosomes derived from pancreatic cancer cells induce insulin resistance in c2c12 myotube cells through the PI3K/akt/foxo1 pathway. Sci. Rep..

[87] Van den Boorn J.G., Schlee M., Coch C., Hartmann G. (2011). Sirna delivery with exosome nanoparticles. Nat. Biotechnol..

[88] Alvarez-Erviti L., Seow Y., Yin H., Betts C., Lakhal S., Wood M.J. (2011). Delivery of sirna to the mouse brain by systemic injection of targeted exosomes. Nat. Biotechnol..

[89] Tran T.H., Mattheolabakis G., Aldawsari H., Amiji M. (2015). Exosomes as nanocarriers for immunotherapy of cancer and inflammatory diseases. Clin. Immunol..

[90] Sercombe L., Veerati T., Moheimani F., Wu S.Y., Sood A.K., Hua S. (2015). Advances and challenges of liposome assisted drug delivery. Front. Pharmacol..

[91] Szebeni J., Moghimi S.M. (2009). Liposome triggering of innate immune responses: A perspective on benefits and adverse reactions. J. Liposome Res..

[92] Raposo G., Stoorvogel W. (2013). Extracellular vesicles: Exosomes, microvesicles, and friends. J. Cell Biol..

[93] Clayton A., Harris C.L., Court J., Mason M.D., Morgan B.P. (2003). Antigen-presenting cell exosomes are protected from complement-mediated lysis by expression of cd55 and cd59. Eur. J. Immunol..

[94] Sun D., Zhuang X., Xiang X., Liu Y., Zhang S., Liu C., Barnes S., Grizzle W., Miller D., Zhang H.G. (2010). A novel nanoparticle drug delivery system: The anti-inflammatory activity of curcumin is enhanced when encapsulated in exosomes. Mol. Ther..

[95] Munagala R., Aqil F., Jeyabalan J., Gupta R.C. (2016). Bovine milk-derived exosomes for drug delivery. Cancer Lett..

[96] Cheng L., Sharples R.A., Scicluna B.J., Hill A.F. (2014). Exosomes provide a protective and enriched source of mirna for biomarker profiling compared to intracellular and cell-free blood. J. Extracell. Vesicles.

[97] Chen C.C., Liu L., Ma F., Wong C.W., Guo X.E., Chacko J.V., Farhoodi H.P., Zhang S.X., Zimak J., Ségaliny A. (2016). Elucidation of exosome migration across the blood-brain barrier model in vitro. Cell. Mol. Bioeng..

[98] Yang T., Martin P., Fogarty B., Brown A., Schurman K., Phipps R., Yin V.P., Lockman P., Bai S. (2015). Exosome delivered anticancer drugs across the blood-brain barrier for brain cancer therapy in danio rerio. Pharm. Res..

[99] Vader P., Mol E.A., Pasterkamp G., Schiffelers R.M. (2016). Extracellular vesicles for drug delivery. Adv. Drug Deliv. Rev..

[100] Bellavia D., Raimondo S., Calabrese G., Forte S., Cristaldi M., Patinella A., Memeo L., Manno M., Raccosta S., Diana P. (2017). Interleukin 3- receptor targeted exosomes inhibit in vitro and in vivo chronic myelogenous leukemia cell growth. Theranostics.

[101] Qi H., Liu C., Long L., Ren Y., Zhang S., Chang X., Qian X., Jia H., Zhao J., Sun J. (2016). Blood exosomes endowed with magnetic and targeting properties for cancer therapy. ACS Nano.

[102] Valadi H., Ekström K., Bossios A., Sjöstrand M., Lee J.J., Lötvall J.O. (2007). Exosome-mediated transfer of mrnas and micrornas is a novel mechanism of genetic exchange between cells. Nat. Cell Biol..

[103] Boelens M.C., Wu T.J., Nabet B.Y., Xu B., Qiu Y., Yoon T., Azzam D.J., Twyman-Saint Victor C., Wiemann B.Z., Ishwaran H. (2014). Exosome transfer from stromal to breast cancer cells regulates therapy resistance pathways. Cell.

[104] Li W., Zhang X., Wang J., Li M., Cao C., Tan J., Ma D., Gao Q. (2017). Tgfβ1 in fibroblasts-derived exosomes promotes epithelial-mesenchymal transition of ovarian cancer cells. Oncotarget.

[105] Ringuette Goulet C., Bernard G., Tremblay S., Chabaud S., Bolduc S., Pouliot F. (2018). Exosomes induce fibroblast differentiation into cancer-associated fibroblasts through tgfβ signaling. Mol. Cancer Res..

[106] Chowdhury R., Webber J.P., Gurney M., Mason M.D., Tabi Z., Clayton A. (2015). Cancer exosomes trigger mesenchymal stem cell differentiation into pro-angiogenic and pro-invasive myofibroblasts. Oncotarget.

[107] Aspe J.R., Diaz Osterman C.J., Jutzy J.M., Deshields S., Whang S., Wall N.R. (2014). Enhancement of gemcitabine sensitivity in pancreatic adenocarcinoma by novel exosome-mediated delivery of the survivin-t34a mutant. J. Extracell. Vesicles.

[108] Mahmoodzadeh Hosseini H., Ali Imani Fooladi A., Soleimanirad J., Reza Nourani M., Mahdavi M. (2014). Exosome/staphylococcal enterotoxin b, an anti tumor compound against pancreatic cancer. J. BUON.

[109] Wahlgren J., De L Karlson T., Brisslert M., Vaziri Sani F., Telemo E., Sunnerhagen P., Valadi H. (2012). Plasma exosomes can deliver exogenous short interfering rna to monocytes and lymphocytes. Nucleic Acids Res..

[110] Zitvogel L., Regnault A., Lozier A., Wolfers J., Flament C., Tenza D., Ricciardi-Castagnoli P., Raposo G., Amigorena S. (1998). Eradication of established murine tumors using a novel cell-free vaccine: Dendritic cell-derived exosomes. Nat. Med..

[111] Viaud S., Terme M., Flament C., Taieb J., André F., Novault S., Escudier B., Robert C., Caillat-Zucman S., Tursz T. (2009). Dendritic cell-derived exosomes promote natural killer cell activation and proliferation: A role for nkg2d ligands and il-15ralpha. PLoS ONE.

[112] Raposo G., Nijman H.W., Stoorvogel W., Liejendekker R., Harding C.V., Melief C.J., Geuze H.J. (1996). B lymphocytes secrete antigen-presenting vesicles. J. Exp. Med..

[113] Besse B., Charrier M., Lapierre V., Dansin E., Lantz O., Planchard D., Le Chevalier T., Livartoski A., Barlesi F., Laplanche A. (2016). Dendritic cell-derived exosomes as maintenance immunotherapy after first line chemotherapy in nsclc. Oncoimmunology.

[114] Dai S., Wei D., Wu Z., Zhou X., Wei X., Huang H., Li G. (2008). Phase i clinical trial of autologous ascites-derived exosomes combined with gm-csf for colorectal cancer. Mol. Ther..

[115] Muller L., Mitsuhashi M., Simms P., Gooding W.E., Whiteside T.L. (2016). Tumor-derived exosomes regulate expression of immune function-related genes in human t cell subsets. Sci. Rep..

[116] Szajnik M., Czystowska M., Szczepanski M.J., Mandapathil M., Whiteside T.L. (2010). Tumor-derived microvesicles induce, expand and up-regulate biological activities of human regulatory t cells (treg). PLoS ONE.

[117] Xiao L., Erb U., Zhao K., Hackert T., Zöller M. (2017). Efficacy of vaccination with tumor-exosome loaded dendritic cells combined with cytotoxic drug treatment in pancreatic cancer. Oncoimmunology.

[118] Su M.J., Aldawsari H., Amiji M. (2016). Pancreatic cancer cell exosome-mediated macrophage reprogramming and the role of micrornas 155 and 125b2 transfection using nanoparticle delivery systems. Sci. Rep..

[119] Bobrie A., Krumeich S., Reyal F., Recchi C., Moita L.F., Seabra M.C., Ostrowski M., Théry C. (2012). Rab27a supports exosome-dependent and -independent mechanisms that modify the tumor microenvironment and can promote tumor progression. Cancer Res..

[120] Ostenfeld M.S., Jeppesen D.K., Laurberg J.R., Boysen A.T., Bramsen J.B., Primdal-Bengtson B., Hendrix A., Lamy P., Dagnaes-Hansen F., Rasmussen M.H. (2014). Cellular disposal of mir23b by rab27-dependent exosome release is linked to acquisition of metastatic properties. Cancer Res..

[121] Datta A., Kim H., McGee L., Johnson A.E., Talwar S., Marugan J., Southall N., Hu X., Lal M., Mondal D. (2018). High-throughput screening identified selective inhibitors of exosome biogenesis and secretion: A drug repurposing strategy for advanced cancer. Sci. Rep..

[122] Ha D., Yang N., Nadithe V. (2016). Exosomes as therapeutic drug carriers and delivery vehicles across biological membranes: Current perspectives and future challenges. Acta Pharm. Sin. B.

[123] Munson P., Shukla A. (2015). Exosomes: Potential in cancer diagnosis and therapy. Medicines.

[124] Kim M.S., Haney M.J., Zhao Y., Mahajan V., Deygen I., Klyachko N.L., Inskoe E., Piroyan A., Sokolsky M., Okolie O. (2016). Development of exosome-encapsulated paclitaxel to overcome mdr in cancer cells. Nanomedicine.

[125] André F., Chaput N., Schartz N.E., Flament C., Aubert N., Bernard J., Lemonnier F., Raposo G., Escudier B., Hsu D.H. (2004). Exosomes as potent cell-free peptide-based vaccine. I. Dendritic cell-derived exosomes transfer functional mhc class i/peptide complexes to dendritic cells. J. Immunol..

